# Biofilm Formation by the Acidophile Bacterium *Acidithiobacillus thiooxidans* Involves c-di-GMP Pathway and Pel exopolysaccharide

**DOI:** 10.3390/genes9020113

**Published:** 2018-02-21

**Authors:** Mauricio Díaz, Matias Castro, Sylvia Copaja, Nicolas Guiliani

**Affiliations:** 1 Bacterial Communication Laboratory, Biology Department, Faculty of Sciences, Universidad de Chile, Ñuñoa 7800003, Santiago, Chile; maujav2004@hotmail.com (M.D.); matiascastrog@gmail.com (M.C.); 2 Organic and Environmental Chemistry Laboratory, Faculty of Sciences, Universidad de Chile, Ñuñoa 7800003, Santiago, Chile; scopaja@uchile.cl

**Keywords:** *Acidithiobacillus*, biofilm, bioleaching, biomining, c-di-GMP, Pel exopolysaccharide, PelD

## Abstract

Acidophile bacteria belonging to the *Acidithiobacillus* genus are pivotal players for the bioleaching of metallic values such as copper. Cell adherence to ores and biofilm formation, mediated by the production of extracellular polymeric substances, strongly favors bioleaching activity. In recent years, the second messenger cyclic diguanylate (c-di-GMP) has emerged as a central regulator for biofilm formation in bacteria. C-di-GMP pathways have been reported in different *Acidithiobacillus* species; however, c-di-GMP effectors and signal transduction networks are still largely uncharacterized in these extremophile species. Here we investigated Pel exopolysaccharide and its role in biofilm formation by sulfur-oxidizing species *Acidithiobacillus* *thiooxidans*. We identified 39 open reading frames (ORFs) encoding proteins involved in c-di-GMP metabolism and signal transduction, including the c-di-GMP effector protein PelD, a structural component of the biosynthesis apparatus for Pel exopolysaccharide production. We found that intracellular c-di-GMP concentrations and transcription levels of *pel* genes were higher in *At*. *thiooxidans* biofilm cells compared to planktonic ones. By developing an *At*. *thiooxidans* Δ*pelD* null-mutant strain we revealed that Pel exopolysaccharide is involved in biofilm structure and development. Further studies are still necessary to understand how Pel biosynthesis is regulated in *Acidithiobacillus* species, nevertheless these results represent the first characterization of a c-di-GMP effector protein involved in biofilm formation by acidophile species.

## 1. Introduction

Biomining is an industrial process in which acidophilic leaching microorganisms including bacteria and archaea are used to recover valuable metals such as copper, cobalt and zinc from low-grade sulfidic ores [1,2]. In addition to its advantaging industrial application, bioleaching naturally occurs in any environment where sulfidic minerals are exposed to both water and oxygen, contributing to water contamination through acid mine/rock drainage (AMD/ARD) generation [3,4]. Microbial leaching activity is increased by bacterial attachment on mineral due to the formation of a thin reaction space between ore and cells [5,6] suggesting that the understanding of molecular events involved in biofilm formation by acidophile species may help to improve biomining and mitigate environmental pollution. Due to the focus on the leaching activities of microorganisms, *Acidithiobacillus* species have been the first and most characterized bioleaching species and were early considered a pivotal player for the biomiming process [7,8]. To date *Acidithiobacillus* genus encompasses seven gram-negative, acidophilic and chemolithoautotrophic species that can only oxidize reduced inorganic sulfur compounds (RISCs) or both ferrous iron and RISCs [9,10,11,12,13]. All *Acidithiobacillus* sp. are capable to form biofilms on mineral surfaces [14,15,16]. Moreover, recent ecological studies pointed out that acidophilic bacterial communities from natural environments frequently occur as biofilms in which *Acidithiobacillus* species are predominant structural members [17,18,19]. Several studies performed with the iron/sulfur oxidizer specie *At. ferrooxidans* revealed that quorum sensing (QS) communication system mediated by acyl-homoserine lactone molecules modulates biofilm formation [20,21,22]. Nevertheless, since sulfur-oxidizing species *At. caldus* and *At. thioooxidans* do not possess any canonical genes for QS [23], it was earlier suggested that biofilm formation should also be regulated by other molecular pathways in the *Acidithiobacillus* species. Indeed, it has been recently reported that the cyclic diguanylic acid (c-di-GMP) pathway is functional and plays an active role in biofilm formation by different *Acidithiobacillus* species [24,25].

The second messenger cyclic diguanylate (c-di-GMP) has emerged as a central metabolite that controls several phenotypes in bacteria, including motility and biofilm formation [26,27]. It is well accepted now that high intracellular levels of c-di-GMP repress motility and stimulate biofilm formation [26,27,28,29,30]. Intracellular levels of c-di-GMP are balanced by the antagonist activities of diguanylate cyclases (DGCs) and phosphodiesterases (PDEs). The synthesis of c-di-GMP is performed by GGDEF domain present in DGC enzymes by using two guanosine triphosphate (GTP) molecules as substrate. C-di-GMP degradation is catalyzed by EAL and HD-GYP domains from two different PDE families [26,27]. Several classes of c-di-GMP effectors that differ in their nature as well as in structural basis for c-di-GMP binding have been described [26,27,31]. Although two different classes of c-di-GMP binding riboswitches have been characterized [32], most of c-di-GMP effectors described so far are proteins. These include the PilZ domain of multimeric protein complexes such as bacterial cellulose synthase (BCS) [33], inactive GGDEF domains such as PelD (see further [29]), inactive EAL domains [34], several transcriptional regulators [26,35,36], ATPases involved in protein secretion [37] and cell-cycle regulators with kinase-phosphatase activity [38]. The inner-membrane protein PelD from *Pseudomonas aeruginosa* was one of the first c-di-GMP effector proteins to be characterized [29]. It is encoded into the seven-gene operon *pelABCDEFG,* the gene products of which are assembled in a multiprotein membrane complex for Pel biosynthesis [39,40]. The binding of c-di-GMP to the cytoplasmic side of PelD increases the glycosil-transferase activity of Pel biosynthetic machinery [29,39]. Pel apparatus architecture and export mechanism have been now deciphered and the structural composition of Pel exopolysaccharide has been characterized [41,42,43]. In *Pseudomonas aeruginosa*, Pel exopolysaccharide is involved in cell aggregation and maintenance of biofilm structure [29,41,44].

In the course for the characterization of c-di-GMP pathways in *Acidithiobacillus* spp. [24,25], comparative genomic studies performed by our group revealed that the complexity of c-di-GMP network differs in *Acidithiobacillus* species [25]. In addition, it was also pointed out that an open reading frame (ORF) coding for the c-di-GMP effector protein PelD and the corresponding *pel*-like operon are present only in the sulfur-oxidizing species *At. thiooxidans* and *At. caldus* while *bcs* operon involved in biosynthesis of cellulose, which is also regulated by c-di-GMP, is present on both sulfur/iron and sulfur oxidizer species [25]. Thus, we hypothesized that Pel exopolysaccharide should play a specific role in biofilm formation by sulfur oxidizing *Acidithiobacillus* species [25]. The purpose of the present work was to challenge this hypothesis. Here we fully characterized a c-di-GMP pathway in *At. thiooxidans*^T^ type strain ATCC 19377 and we investigated the role of Pel exopolysaccharide on biofilm formation by this extremophile microorganism. By high-performance liquid chromatography (HPLC) and quantitative polymerase chain reaction (qPCR) experiments we demonstrated that intracellular c-di-GMP concentrations and transcription levels of *pel* genes are increased in *At*. *thiooxidans* biofilm cells compared to planktonic ones. In correlation with both results, we demonstrated that Pel exopolysaccharide is involved in biofilm structure by developing an *At*. *thiooxidans* Δ*pelD* null-mutant strain. Finally, this work provides the first evidence that the c-di-GMP pathway and Pel exopolysaccharide are both involved in biofilm formation by acidophilic bacteria.

## 2. Materials and Methods

### 2.1. Strains, Plasmids, Primers and Growth Conditions

Strains, plasmids and primers used in this work are described in Tables S1 and S2. *At. thiooxidans* ATCC 19377^T^ strain was grown at 30 °C in modified Mackintosh (MAC) medium [45] pH 4.5 supplemented with different energetic substrates: 5% *w*/*v* elemental sulfur (S°; prills and coupons); 20 mM thiosulfate (Na_2_S_2_O_3_), 10 mM tetrathionate (K_2_S_4_O_6_). Solid medium was obtained by adding 1 mM MgSO_4_, 8 mg/L Bromocresol Green and 0.88% phytagel (*w*/*v*). *At*. *thiooxidans*^T^ null-mutant strain Δ*pelD* was grown in selective media with 100–200 μg/mL kanamycin. *Escherichia coli* and *Salmonella enterica* serovar Typhimurium strains were grown at 37 °C in Luria-Bertani (LB) medium (1% Triptone, 0.5% yeast extract, 0.5% NaCl) pH 7.0 and agar (1.5% *w*/*v*) was added for solid medium. Selective media for *E. coli* strains were supplemented with ampicillin (100 μg/mL), trimetroprin (50 μg/mL), chloramphenicol (20 μg/mL) or kanamycin (30 μg/mL). The mating medium for conjugation was made by adding 0.5 mM d-Glucose, 0.05% yeast extract and 50 μM diaminopimelic acid into solid thiosulfate growth medium.

### 2.2. Bioinformatic Analysis

*At*. *thiooxidans*^T^ draft genome AFOH01000001 [46] was obtained from National Center for Biotechnology Information (NCBI) Database. Candidate genes for proteins with GGDEF, EAL, HD-GYP and c-di-GMP effectors domains were predicted using the basic local alignment search tool (BLAST) as previously described [25]. Annotation results were visualized with Artemis software [47]. Protein domains were identified using Pfam [48] and Prosite [49]. Transmembrane domain predictions were done by TMHMM Server [50]. The functionality of identified domains was predicted using ClustalO algorithm [51].

### 2.3. qPCR Experiments

*At*. *thiooxidans*^T^ cells were grown in 200 mL of medium with sulfur, thiosulfate or tetrathionate for five days. Planktonic cells were collected by centrifugation at 6000× *g* for 10 min. Biofilm cells were separated from solid sulfur by incubation with 0.05% Triton X-100 and collected by centrifugation [24]. Total RNA was extracted from both cell sub-populations as previously described, incubated with DNase I at 37 °C for 1 h and purified by phenol-chloroform treatment [24]. The complementary DNA (cDNA) was synthetized from 1 µg of total RNA by using reverse transcriptase and random primers. Then, cDNA was diluted 1/30 with nuclease-free water and used as template for qPCR experiments. Specific primers were designed to analyze transcriptional levels of the *pelA*, *pelD* and *wcaG* genes. *16S rDNA* and *map* were used as housekeeping genes for data normalization [52].

### 2.4. Nucleotide-Enriched Fraction Extraction and c-di-GMP Analysis

Nucleotide-enriched fractions were extracted from late-exponential growing cells from sulfur, thiosulfate or tetrathionate cultures. Sulfur cultures were separated in two independent cell populations: planktonic and biofilm cells [24]. The extraction was realized by hot lysis and HClO_4_ treatments [53]. HPLC analysis was performed by a HPLC coupled to photodiode array detector (Waters 1525, 2996) (Waters, Milford, MA, USA) using a 15 cm × 3 mm SUPELCOSIL LC-18-DB C18, 3 μm particle size, Reverse Phase Column (SIGMA, Saint Louis, MO, USA). The liquid chromatography (LC) system consisted of degasser (Waters), binary pump (Waters) and oven (Waters). The mobile phase was methanol (A) and water pH 6.0 (6 mM KH_2_PO_4_) (B). Elution conditions were 5 min at 100% B, 15 min linear gradient from 100% B to 20% A and 80% B and finally 10 min with a gradient from 20% A and 80% B to 100% B with a constant flow of 0.4 mL/min. The temperature was set at 30 ± 3 °C. The injection volume was 20 µL. The calibration curve was performed with synthetic c-di-GMP (BIOLOG, Hayward, CA, USA) in a range of 6.9–552 ng (10–800 pmol) for injection. Signal signatures were identified by coincidence of retention times of 12.531 ± 0.295 min and comparison of absorption spectra at 252.4 nm. Data were expressed as pmol c-di-GMP and normalized against cellular wet weight.

### 2.5. Diguanylate Cyclase Activity

Heterologous complementation assays in *Salmonella* strain defective in DGC activity were performed as previously described [25]. Briefly, several *At. thiooxidans*^T^ genes encoding for different proteins with GGDEF domains were amplified from genomic DNA by PCR and cloned into pBAD24 plasmid. *Salmonella enterica* serovar Typhimurium AdrA1f strain was electrotransformed with pBAD24 recombinant plasmids harboring *At. thiooxidans*^T^ genes and DGC activity was evaluated by congo red binding assay [25].

### 2.6. Construction and Selection of a At. thiooxidans^T^ ΔpelD Null Mutant Strain

*At. thiooxidans*^T^ Δ*pelD* null mutant strain was constructed as described in Castro et al. [25]. First, a Δ*pelD* suicide plasmid was produced by molecular engineering. Briefly, two different 800 bp DNA fragments carrying 5′ and 3′ extremes of *pelD* gene were obtained by PCR. Primers harbored specific restriction sites for cloning and fragment production in pGEM-T vector (Promega, Madison, WI, USA). *Kan^R^* gene was released from plasmid pSKM2 [54] by using restriction enzymes HindIII and XmaI. The plasmid pOT was digested by restriction enzymes SacI and XbaI and then dephosphorylated with alkaline phosphatase. All DNA restriction-fragments were separated by electrophoresis, recovered from agarose gels and quantified. Ligations were performed with T4 DNA ligase at 4 °C overnight and ligation product was transformed into chemocompetent *E*. *coli* JM109 cells. Recombinant cells were selected on solid medium supplemented with ampicillin and kanamycin resistance. Suicide plasmid pOT-*pelD*::*kan^R^* was checked by restriction analysis and sequencing (MACROGEN, Seoul, Korea).

For conjugation assays, *At*. *thiooxidans*^T^ cells were grown on thiosulfate to reach a 10^8^ cells/mL density. *E*. *coli* HB101 strain carrying pOT-*pelD*::*kan^R^* and pR388 [54] plasmids was grown overnight in selective LB medium to inoculate 50 mL of liquid mating medium with corresponding antibiotics. Then *E. coli* cells were grown overnight at 37 °C and collected. Both *At. thiooxidans* and *E. coli* cells fractions were separately washed twice with modified MAC medium (pH 4.5) and collected by centrifugation (6000× *g*, 10 min). Both cell suspensions were homogeneously mixed (cell ratios 1:1) and 100 μL of this cellular mix was spotted on a sterile polycarbonate filter. Inoculated filters were gently located over a solid mating medium and incubated for 5 days at 30 °C. Then filters were picked off and incubated for 7 days at 30 °C in liquid MAC medium with thiosulfate (20 mM) and kanamycin (200 μg/mL). Finally, cultures were diluted and 100 μL of 10^−4^, 10^−5^ and 10^−6^ dilutions were plated on selective solid MAC medium (200 μg/mL kanamycin) and incubated at 30 °C for several days until the appearance of colonies.

Colonies were first analyzed by PCR with specific *kan^R^* primers. They were picked up and re-suspended in 10 μL of MAC medium pH 4.5. The cell suspension was spotted in solid selective MAC medium for growth. A fraction of the grown spot was collected and re-suspended in 100 μL of a 25 mM Tris-HCl (pH 7.5), 3 mM KCl solution. The cell suspension was heated at 100 °C and centrifuged to eliminate cell debris. Thus, the cell lysate was used as a template for the amplification of *16S rDNA*, *kan^R^* and *pelD* genes by PCR. Finally, PCR products were run in 1.5% *w*/*v* agarose gels in TAE buffer (40 mM Tris, 20 mM acetic acid, and 1 mM EDTA, pH 8.2) and electrophoretic pattern were compared to wild type strain. Positive colonies were finally analyzed by Southern blot analysis using specific *pelD* and *kan^R^* labeled with digoxigenin [25]. Genomic DNA (10 µg) from mutant and wild type strain or control plasmids (1 µg) were digested with *Sph*I or *Bam*HI for *pelD* or *kan^R^* probes, respectively. All DNAs were run in 1% agarose gel using TBE buffer 1× (89 mM Tris, 89 mM boric acid, 2 mM EDTA, pH 7.6). The SSC buffer 10× (1,5 M NaCl, 0.15 M sodium Citrate, pH 7.2) was used to transfer DNA fragments from gel to nylon membrane. Final concentration for probes hybridization was 25 ng/mL and developing was done according to manufacturer’s instructions (Roche, Basel, Switzerland).

### 2.7. Visualization of At. thiooxidans^T^ Biofilms

*At*. *thiooxidans*^T^ cells were grown in MAC medium with sulfur (Merck, Darmstadt, Germany) prills and coupons as solid energetic substrate. Sulfur coupons were used for biofilm visualization. Thus, colonized coupons were extracted at different incubation times for 5 days, washed once with aqueous H_2_SO_4_ pH 2.0, once with 50 mM Tris-HCl, 1 mM EDTA, pH 7.5 and once with bidistilled water to remove all the remaining planktonic cells. Afterwards cells were fixed overnight with 4% formaldehyde. These coupons were critical point dried, coated with gold and analyzed by Scanning Electron Microscopy (SEM) (LEO 1530VP, LEO Electron Microscopy Inc., Thornwood, NY, USA), as previously described [25].

### 2.8. Quantification of Extracellular Polymeric Substances 

Cells were incubated in 500 mL of MAC medium with sulfur for 5 days. Sulfur-prills colonized with cells were collected and washed twice with 10 mM KH_2_PO_4_ pH 4.5 to eliminate any remaining planktonic cells. Then prills were vortexed with 0.05% Triton X-100 in 10 mM KH_2_PO_4_ pH 4.5 for 10 min twice to release extracellular polymeric substances (EPS) and attached cells. Sulfur debris and released cells were removed by centrifugation. Supernatant was recollected and EPS were recovered by incubation with ethanol and ultracentrifugation at 4 °C (100,000× *g*, 1 h). EPS sediment was re-suspended in 300 µL of 20 mM Tris-HCl, 1 mM EDTA pH 7.4 and stored at −20 °C. Carbohydrates determination was done using the Dubois method [55], while protein content was measured using the Bicinchoninic Acid method [56].

## 3. Results

### 3.1. Acidithiobacillus thiooxidans^T^ Possesses a Functional c-di-GMP Pathway

The bioinformatical analysis of *At*. *thiooxidans*^T^ genome allowed the identification of twenty-five ORFs coding for putative DGCs and PDEs proteins that could be involved in metabolism of c-di-GMP (Figure S1). Twelve of them encode for proteins with both GGDEF/EAL domains (*ATHIO_RS1701500*, *ATHIO_RS1815000*, *ATHIO_RS0108445*, *ATHIO_RS1708000*, *ATHIO_RS0100160*, *ATHIO_RS0110800*, *ATHIO_RS179300*, *ATHIO_RS0102030*, *ATHIO_RS0113350*, *ATHIO_RS1813000*, *ATHIO_RS0104240*, *ATHIO_RS0114625*), 9 for putative DGC with single GGDEF (*ATHIO_RS1657500*, *ATHIO_RS0108455*, *ATHIO_RS0108485*, *ATHIO_RS1689500*, *ATHIO_RS1835500*, *ATHIO_RS1838000*, *ATHIO_RS0107955*, *ATHIO_RS0116120*, *ATHIO_RS1781000*), 3 for putative PDE with single EAL domains (*ATHIO_RS0108450*, *ATHIO_RS1750000*, *ATHIO_RS0113355*) and one for a putative PDE with HD-GYP domain (Figure S2). Moreover, multiple alignment analysis of all these c-di-GMP metabolic domains showed that most of them (15/21 GGDEF domains, 14/15 EAL domains and 1/1 HD-GYP domain) possess all amino acids required for catalytic activity [21]. Different sensor domains such as GAF and PAS have been also predicted inside the amino acid sequence of some of these proteins (17/25) (Figure S2) suggesting that the global intracellular level of c-di-GMP in *At*. *thiooxidans*^T^ is modulated by different environmental factors. In addition, fourteen ORFs coding c-di-GMP effector proteins were also identified (Figure S1). Nine of them encode for proteins with PilZ domains including five Type IV pilus assembly proteins (Table S3), one for a PelD-like protein, two for putative transcriptional regulators FleQ, one for an ATPase with MshEN domain and one for a YajQ-like protein. Interestingly, several assigned functions (BlastP Hit) for these putative c-di-GMP effectors were related to biofilm formation such as pilus assembly (*ATHIO_RS16400*, *ATHIO_RS0105675*, *ATHIO_RS0109125*, *ATHIO_RS0109755*, *ATHIO_RS0110790* and *ATHIO_RS0114620*), motility regulation (*ATHIO_RS0108750*) and synthesis of exopolysaccharides such as cellulose (*ATHIO_RS0101475*) and Pel (*ATHIO_RS018015*). Finally, reverse transcriptase (RT-)PCR experiments using total RNA obtained from planktonic cells grown on sulfur revealed that most of these genes are transcribed (Figure S1).

**Figure 1:**
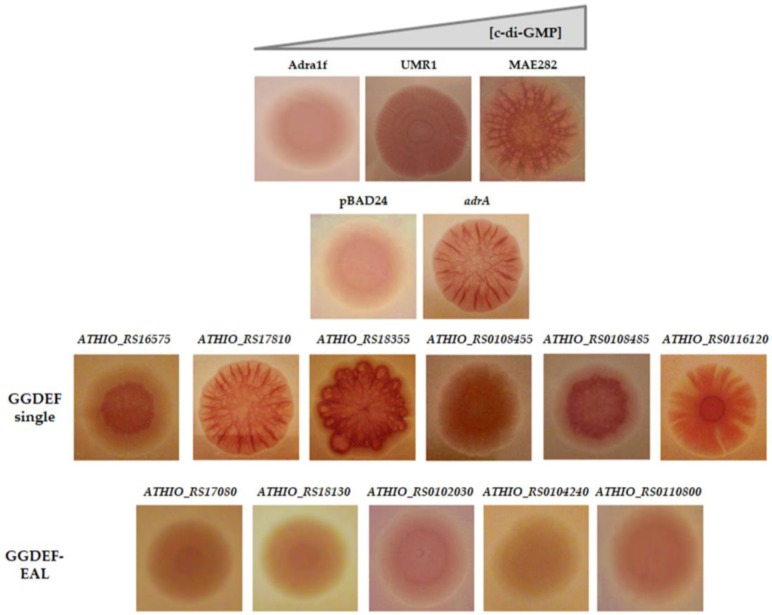
Heterologous complementation in *Salmonella enterica* serovar Typhimurium strain AdrA1f of diguanylate cyclase activity from single GGDEF and GGDEF/EAL encoding genes of *Acidithiobacillus thiooxidans*^T^. The rdar (rough, dry and red) morphotype [57], which is induced by diguanylate cyclase (DGC) activity was analyzed on congo red agar plates and compared to wild type (UMR1), DGC null-mutant (AdrA1f), DGC complemented (p*adrA*), negative control (pBAD24 without insert) and a phosphodiesterase null-mutant (MAE 282) strains.

**Figure 2:**
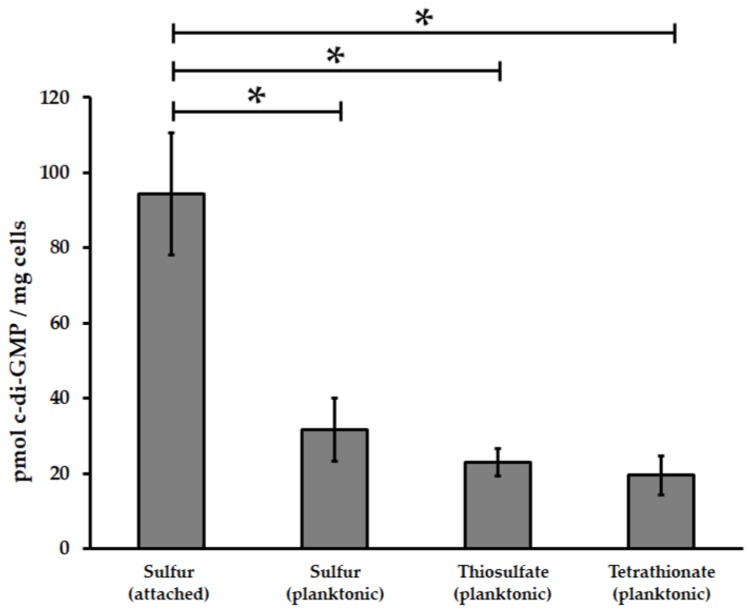
Comparative analysis of c-di-GMP levels from biofilm and planktonic *At. thiooxidans*^T^ cells grown in different energetic substrates. The nucleotide-enriched extracts were analyzed by high-performance liquid chromatography (HPLC) coupled to photodiode array detector. Values represent the average of tree independent experiments ± standard deviation. Significant differences made by a one-way analysis of variance (ANOVA) test (*p* < 0.05) are noted (*).

In order to assess DGC activity, 11 GGDEF domains encoding genes from *At*. *thiooxidans*^T^ including six putative DGC enzyme with single GGDEF domain and five with both EAL/GGDEF domains (Figure S1) were cloned in *Salmonella enterica* serovar Typhimurium AdrA1f strain. Congo red phenotypic assays only revealed strong positive DGC activity, based in the DGC activity-induced rdar (rough, dry and red) morphotype of the colony [57], for proteins with single GGDEF domains (Figure 1). In addition, to evaluate the relationship between c-di-GMP pathway and biofilm formation by *At*. *thiooxidans*^T^, intracellular c-di-GMP levels were measured in different cell sub-populations by HLPC. Planktonic populations were obtained from cells grown in thiosulfate, tetrathionate and elemental sulfur, while biofilm cells were obtained from sulfur cultures. In our experimental conditions, intracellular concentrations of c-di-GMP were 3.5-fold higher in five days sulfur-biofilm cells compared to planktonic cells grown in sulfur, thiosulfate or tetrathionate (Figure 2). Both results clearly indicate that c-di-GMP pathway is functional in *At*. *thiooxidans*^T^. In addition, they revealed that attachment of *At*. *thiooxidans*^T^ cells to solid energetic substrate is directly related to an increase of intracellular concentration of c-di-GMP.

### 3.2. Pel Genes Are Overexpressed in At. thiooxidans^T^ Attached Cells

**Figure 3:**
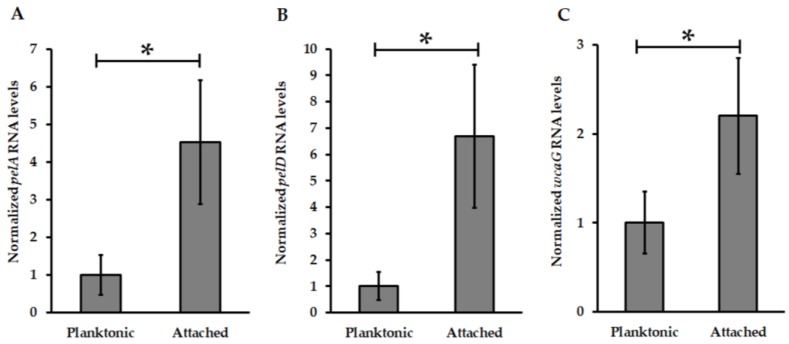
Transcription levels analysis of Pel biosynthesis machinery encoding genes from *At*. *thiooxidans*^T^. Transcript levels of *pelA* (**A**), *pelD* (**B**) and *wcaG* (**C**) genes were measured by quantitative polymerase chain reaction (qPCR) and then normalized using DNA *16S* and *map* genes. Values represent the average of four independent experiments ± standard deviation. Significant differences made by a one-way ANOVA test (*p* < 0.05) are noted (*).

Among the 14 putative c-di-GMP effectors proteins identified in the available draft genome of *At. thiooxidans*^T^, a *pelD* orthologous gene was found. Moreover, the analysis of genomic context revealed that this *pelD*-like is located inside a putative *pel* operon which is present in other *At. thiooxidans* strains [58]. Interestingly, *pel* operon structure from *At. thiooxidans*^T^ is similar to *pel* operon from *At. caldus* including for the presence downstream *pelG* of an additional *wcaG* gene coding for an enzyme with uridine diphosphate (UDP)-glucose-4-epimerase activity (Figure S3). As previously noted [25,58,59], *pel*-like operon is harbored by *Acidithiobacillus* species that can oxidize only RISC suggesting a specific role in biofilm formation by *At. caldus* and *At. thiooxidans*. To determine the role of Pel biosynthesis apparatus, transcription levels of *pelA*, *pelD* and *wgcA* genes encoding for the deacetylase PelA, the c-di-GMP effector protein PelD and an UDP-Glucose-4-epimerase respectively were measured by qPCR experiments using total RNA obtained from planktonic and biofilm cells of *At. thiooxidans*^T^. Compared to planktonic cells, transcription levels of *pelA*, *pelD* and *wcaG* genes were increased in *At. thiooxidans* biofilm cells 4.5-, 6.7- and 2.2-fold, respectively (Figure 3).

### 3.3. The PelD Null Mutation Changes At. thiooxidans^T^ Biofilm Structure on Sulfur Surface

To better understand the function of Pel exopolysaccharide in biofilm formation by *At. thiooxidans*, the construction of ∆*pelD* null-mutant strain was challenged. A suicide vector harboring a kanamycin (*kan^R^*) cassette and 5′ and 3′ ends of *At*. *thiooxidans*^T^ *pelD* gene was constructed and introduced by conjugation in *At*. *thiooxidans*^T^. Two hundred recombinant colonies were analyzed by PCR experiments against *pelD*, *kan^R^* and DNA *16S* genes to discriminate single recombinant (*pelD*+, *kan^R^*+, DNA 16S+) and double recombinant (*pelD*−, *kan^R^*+, DNA 16S+) strains. Four clones were selected as double recombinants (Figure S4) and Southern Blot analysis were performed to corroborate the gain of ∆*pelD* null-mutant strain of *At*. *thiooxidans*^T^ (Figure S5).

EPS production from sulfur-grown cells of wild type and ∆*pelD* strains was examined. As expected for the absence of Pel exopolysaccharide, a six fold decrease of total carbohydrates quantity was observed for ∆*pelD* S^0^-attached cells compared to wild type (Table 1). Surprisingly, the measurement of total protein fraction revealed an increase of 33.6% in ∆*pelD* cells compared to wild-type (WT) strain (Table 1).

**Figure 4:**
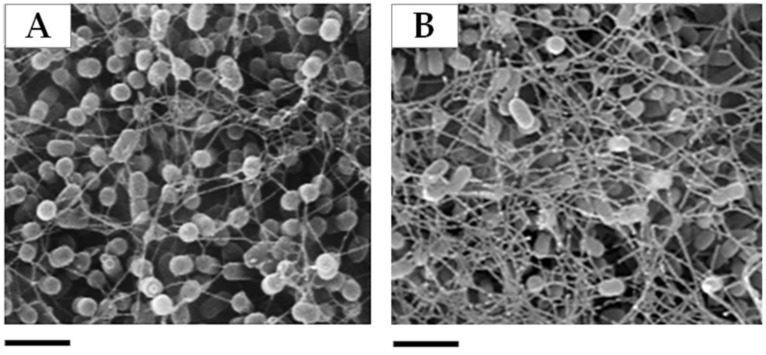
Biofilm structure of *At*. *thiooxidans* is affected by *pelD* deletion. Compared to wild type strain *At*. *thiooxidans*^T^ (**A**), ∆*pelD* null-mutant strain of *At*. *thiooxidans*^T^ (**B**) overexpressed a filamentous structure. Bars represent 2 µm.

In addition, 5-days old biofilms developed on S°-coupons surface by *At. thiooxidans* Δ*pelD* and WT cells were visualized separately by SEM microscopy. As shown in Figure 4, Δ*pelD* null-mutant strain overexpressed a filamentous structure compared to wild type. Both results strongly indicated that biofilm composition and structure of *At. thioooxidans* ∆*pelD* null-mutant strain are modified in comparison to WT strain. Although further studies are still necessary to characterize the nature of this filamentous structure, the EPS quantification result suggests that it could be proteinaceous.

## 4. Discussion

Insights into the c-di-GMP pathway in *Acidithiobacillus* genus have recently started to emerge [24,25]. These studies indicate that c-di-GMP signaling is a widespread pathway into *Acidithiobacillus* genus [25]. As it occurs for *At. ferrooxidans* [24] and *At. caldus* [25], higher c-di-GMP levels were observed in attached cells of *At. thiooxidans*^T^ compared to planktonic cells (Figure 2), supporting that the c-di-GMP pathway regulates biofilm formation by this acidophilic leaching species. With twenty-five genes encoding proteins with GGDEF alone (9), EAL alone (3), both GGDEF/EAL (12) and HD-GYP (1) domains as well as fourteen genes encoding putative effector proteins, *At. thiooxidans*^T^ possesses the c-di-GMP pathway with the highest complexity currently known among species of this genus [24,25]. Moreover, *At. thiooxidans* has a HD-GYP domain encoding gene which was transcribed (Figure S1) suggesting that unlike *At. ferrooxidans* and *At. caldus*, PDE activity should be generated by both EAL and HD-GYP domains in this bacterial species. To date no c-di-GMP riboswitches [32] have been identified in *Acidithiobacillus* genomes [60] suggesting that c-di-GMP effector proteins are the predominant way for c-di-GMP signal transduction in this genus. Indeed, this work allowed the identification of five different proteins families for c-di-GMP effectors in *At. thiooxidans* (Table S3). Altogether, these data suggest that the c-di-GMP pathway signaling has a specific molecular network *At. thiooxidans*, even different to other sulfur-oxidizing species such as *At. caldus*.

The MshE ATPase from *Vibrio cholerae* has been recently characterized as a new c-di-GMP effector protein involved in the biosynthesis and function of Type IVa MshA pili which is a relevant extracellular and adhesive appendage for initial attachment to surfaces by bacterial cells [32]. The binding of c-di-GMP occurs through the MshE N-terminal domain that is the longest nucleotide-binding motif identified yet [61]. Here we identified a *mshE* orthologue (*ATHIO_RS0109755*) that encodes a MshE ATPase-like with a canonical MshE N-terminal domain that was transcribed in *At. thiooxidans* cells grown on elemental sulfur (Figure S1). In addition, the mining of *At. thiooxidans*^T^ genome sequence revealed the presence of several type IV pilin-like protein encoding genes (Table S3). Thus, it is possible to hypothesize that the initial attachment to solid energetic substrates by *At. thiooxidans*^T^ may be regulated by c-di-GMP pathway through MsHE/Type IVa pilin system.

**Figure 5:**
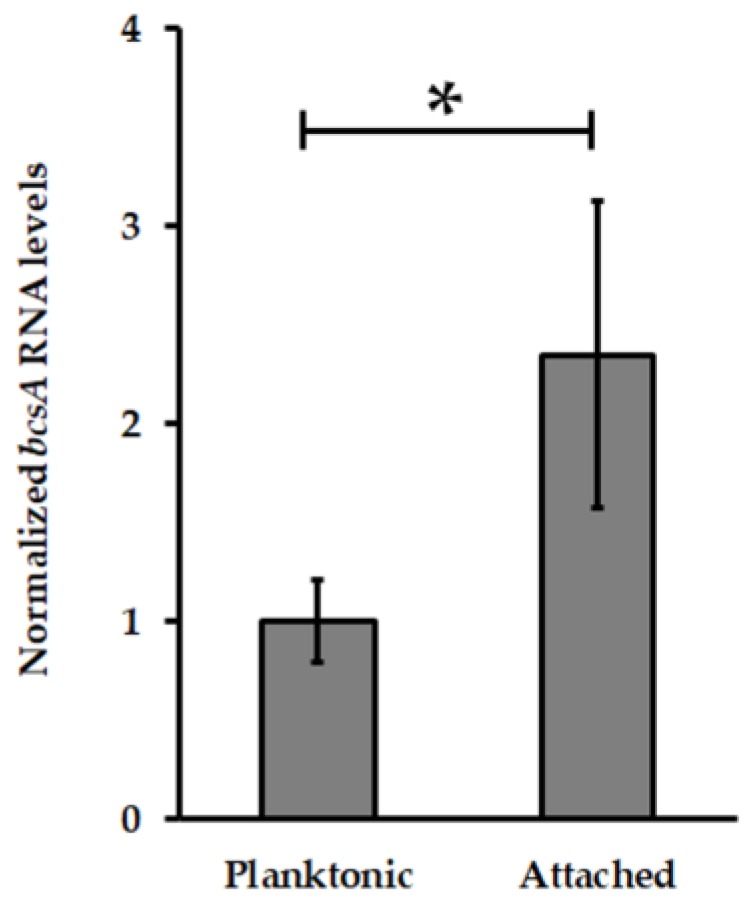
Transcription levels analysis of BcsA (cellulose synthase catalytic subunit) encoding gene from *At*. *thiooxidans*^T^. Transcript levels of *bcsA* were measured by qPCR and then normalized using DNA *16S* and *map* genes. Values represent the average of four independent experiments ± standard deviation. Significant differences made by a one-way ANOVA test (*p* < 0.05) are noted (*).

FleQ was first characterized in *P. aeruginosa* as a transcriptional regulator for genes related to flagellar-based motility and mucin adhesion [62]. Then it was identified as a c-di-GMP effector involved in Pel exopolysaccharide biosynthesis [35] and its pivotal role for production of biofilm matrix components such as cellulose as well as the regulation of flagellar motility has been well documented [63,64,65,66]. Most of these c-di-GMP-regulated machineries have been identified in *At. thiooxidans*. As reported for *At. caldus* [25], *At. thiooxidans* also possesses a *bcsAB* operon involved in cellulose biosynthesis. Moreover, the transcription level of *bcsA* gene (*ATHIO_RS0101475*), encoding for the cellulose synthase catalytic subunit (BcsA), involved in the regulation of cellulose biosynthesis through the binding of c-di-GMP to its PilZ domain [28,33,67,68] was increased in *At. thiooxidans* S^0^-biofilm cells compared to planktonic ones (Figure 5) suggesting that cellulose participates to the biofilm architecture in this *Acidithiobacillus* species. In addition, a flagellar encoding operon has been also identified in *At. thiooxidans* [46]. Thus, the identification of two transcribed *fleQ*-like genes in *At. thiooxidans*^T^ (Figure S1) strongly points out that in addition to Pel exopolysaccharide, c-di-GMP levels could regulate the biosynthesis of cellulose and flagella in this *Acidithiobacillus* species. Interestingly, *At. ferrooxidans* is a primary colonizer that increases mineral colonization by sulfur-oxidizing species such as *At. thioooxidans* [69] but it does not have the genetic capacity to produce Pel exopolysaccharide, cellulose and flagellum (Table S3). This suggests that capsular exopolysaccharide whose expression is induced in *At. ferrooxidans* cells attached to mineral [14] and/or a not yet identified matrix component have to play a pivotal role for biofilm formation by this iron/sulfur-oxidizing species.

Due to the huge difficulties for genetic manipulation of *Acidithiobacillus* spp., very few (up to five) knockout mutant strains have been reported [54,70,71,72,73]. Recently, an *Acidithiobacillus* DGC-defective mutant strain has been developed by our research group [25]. This *At. caldus DGC* null-mutant strain revealed that c-di-GMP pathway is directly involved in the regulation of motility and adherence to sulfur surfaces in this *Acidithiobacillus* species. However, the identification of molecular players connecting the decreased amounts of c-di-GMP intracellular levels with phenotypical observations is still an open question. Castro et al. [25] hypothesized that Pel exopolysaccharide should be involved in biofilm formation by *Acidithiobacillus* species that can only oxidize RISCs, namely *At. caldus* and *At. thiooxidans* and this hypothesis was tested. The results obtained from EPS quantification (Table 1) and qPCR assays demonstrating that transcription levels of several genes from *pel* operon including the c-di-GMP effector protein encoding gene *pelD* were enhanced in biofilm cells compared to planktonic cells (Figure 3), as well as the mutagenesis and SEM experiments allowing the visualization of a filamentous structure which was overexpressed in Δ*pelD* null-mutant strain compared to wild type strain (Figure 4) clearly revealed that Pel exopolysaccharide is involved in biofilm architecture developed by *At. thiooxidans*. Because the proteinaceous fraction was increased in Δ*pelD* null-mutant strain (Table 1) we infer that these filamentous compounds should be proteinaceous. It has been reported that flagellum and amyloid curli fibers are involved in macrobiofilm architecture developed by *E. coli* [74,75]. Bioinformatic search on *At. thiooxidans*^T^ genome sequence revealed that canonical *csgD*-like and *csgBAC*-like genes involved in the synthesis of the most characterized amyloid fibers are absent from all *Acidithiobacillus* genomes. This suggests that *Acidithiobacillus* species either do not produce amyloid fibers or produce some yet uncharacterized amyloid fibers related to its acidophilic lifestyle. In contrast, predicted genes for flagella formation has been identified in *At. thiooxidans*, *At. caldus* and *At. ferrivorans* but not in *At. ferrooxidans* [17,46]. On the other way, because we demonstrated that the transcription level of *bcsA* gene are increased in biofilm cells (Figure 5), this overproduced filamentous structure could be a mesh of flagella used as scaffold for the formation of cellulose filaments as it has been reported in *E. coli* macrocolony biofilm [75]. However further studies are still necessary to decipher the nature of this filamentous structure and the molecular network involved in its biosynthesis.

## Figures and Tables

**Table 1:** Quantification of carbohydrates and proteins levels into *Acidithiobacillus thiooxidans*^T^ S°-attached cells obtained from wild type ATCC 19377 and Δ*pelD* strains.

	ATCC 19377	Δ*pelD*
Carbohydrates (µg/g cells)	1596.80 ± 67.71	272.92 ± 45.88
Proteins (µg/g cells)	245.58 ± 58.33	331.42 ± 52.13

## Supplementary Materials

The following are available online at http://www.mdpi.com/2073-4425/9/2/113/s1, Table S1: Strains and plasmids used in this work, Table S2: Primers used in this work, Table S3: Type IV pilin-like protein and pili apparatus subunits encoding genes in *At. thioooxidans*^T^. Table S4: Putative molecular players for biofilm architecture identified in two iron/sulfur- and two sulfur-oxidizing species of *Acidithiobacillus*, Figure S1: RT-PCR analysis of c-di-GMP metabolism and effectors encoding genes identified in *At. thiooxidans*^T^, Figure S2: Domain organization of *At*. *thiooxidans* ATCC 19377 proteins involved in c-di-GMP metabolism, Figure S3: Comparative analysis of *pel* operon structures, Figure S4: PCR analysis of *At*. *thiooxidans* ATCC 19377 and the four double recombinant Δ*pelD* mutant strains to check double-recombination, Figure S5: Southern blot analysis of *At*. *thiooxidans* ATCC 19377 and the four double recombinant Δ*pelD* mutant strains.
