# Supplementary material for: Biofilm Formation by the Acidophile Bacterium Acidithiobacillus thiooxidans Involves c-di-GMP Pathway and Pel exopolysaccharide
Source: Genes (Basel). 2018 Feb 21;9(2):113. doi: 10.3390/genes9020113 (PMC5852609; doi:10.3390/genes9020113)
Supplement: Supplementary file 1 [file genes-09-00113-s001.pdf]

Supplementary Material for Biofilm Formation by the Acidophile Bacterium *Acidithiobacillus thiooxidans* Involves c-di-GMP Pathway and Pel exopolysaccharide

Mauricio Diaz, Matias Castro, Sylvia Copaja and Nicolas Guiliani

Table S1. Strains and plasmids used in this work.

Strains		Description [1]
Acidithiobacillus thiooxidans		
	ATCC 19377	Type strain, Wild Type
	ATCC 19377-Δ*pelD*	This work: ATCC 19377, *pelD*::*kan*, Kan^R
Escherichia coli		
	JM109	Amplification of pGEM-T Easy constructions
	LMG194	Reservoir of pBAD24
	HB101	Strain for conjugation
Salmonella enterica **serovar Typhimurium**		
	UMR1	WT
	AdrA1f	UMR1 Δ*adrA*, Kan^R
	MAE282	UMR1 Δ*yciR*, Cm^R
**Plasmids**		
	pGEM-T Easy	IPTG-inducible amplification vector, Amp^R
	pBAD24	Arabinose-inducible expression vector, Amp^R
	pOT	Suicide plasmid, Amp^R
	pSKM2	Source of *kan* gene, Kan^R
	pOT-*pelD*	pOT with cloned *pelD* gene, Amp^R
	pOT-*pelD*::*kan*^{R}	Suicide plasmid for *At. thiooxidans*, Amp^R, Kan^R
	pR388	Helper plasmid, Tmp^R

[1] Amp^R, Kan^R, Cm^R and Tmp^R mean Ampicillin, Kanamycin, Chloramphenicol and Trimetropin resistance, respectively.

Table S2. Primers used in this work

Name	Sequence 5′-3′ [1]
*pelA1*_F	CCGATTGCCGCAGTTATTTATT
*pelA1*_R	GCTGTCTTGATGGCTTTGATG
*pelD*_F	CACAAGTTGGCATCCTGGTTCGTT
*pelD*_R	CATGCTGCCTGCGAAAGGTAACAA
16S-F3	ATGGCCTTTATGTCCAGGGCTACA
16S-R3	AATCCGAACTACGACGCGCTTTCT
Ath_map_f	GGACCGGATTTGTCACGATTA
Ath_map_r	GACGTGGTTGAGGGAAATACA
BcsAF	TTGTGACCACATACCAACCAGGGA
BcsAR	TGGGCCTCTTGGCATATCCAGATT
AtthioxUDP F	GAACTTGTCAATGCGCCATC
AtthioxUDP R	GGCCAGCAATAAATCCTGAATAC
T7	AATACGACTCACTATAG
SP6	ATTTAGGTGACACTATAG
*pelD*5'-F	ACCGAGCTCAGGAATCTTGATTCCCATTTTG
*pelD*5'-R	ACCAAGCTTAAATATTTCCAGGCTCCGTATCTC
*pelD*3'-F	ACCCCCGGGTGATTTTCATACTGGTCCTTATG
*pelD*3'-R	ACCTCTAGAGCATTCAAGTTTATCAGTAATTC
KanR_div_5'	GCTTTCTACGTGTTCCGCTTC
KanR_div_3'	TCTATGAAAGGTTGGGCTTC
pOTsec_F	CAGCTATGATGCAGGCAAAC
pOTsec_R	CAGTGAGCGCAACGCAATTA
ATT_F	GGGTGCTAATANCGCCTGCG
907R	CCGTCAATTCMTTTGAGTTT
probe_*At_pelD*_F	CCACCCGCTTTCGACAATTA
probe_*At_pelD*_R	CAGGATGCCAACTTGTGGTA
probe_Kan_R_F	GCTTACATGGCGATAGCTAGAC
probe_Kan_R_R	GAACTCGTCAAGAAGGCGATAG
AthiA1_00157/FN	ACCGCTAGCTAAGGAGGCGAGATCATGGGTGCCTGGC
AthiA1_00157/RH	ACCAAGCTTCAATGATGATGATGATGATGATGATGGTTTCTGGGCTGTAAACGCATAGCC
AthiA1_07018/FN	ACCGCTAGCTAAGGAGGGGAAAGCATGTCCAGTCCATTC
AthiA1_07018/RP	ACCCTGCAGTCAATGATGATGATGATGATGATGATGACTGTCGCTTTCAGGATGAATG
24-7 F/NheI	ACCGCTAGCTAAGGAGGCCCATCCATGGGCGTTTCTCTA
24-7 R/HindIII	ACCAAGCTTCAATGATGATGATGATGATGATGATGAGCTTGTAGGGGCGTATCC
AthiA1_08189/FN	ACCGCTAGCTAAGGAGGCATCATAATGCTTATTGAAGCTTTTTTGCCG
AthiA1_08189/RS	ACCGCATGCTCAATGATGATGATGATGATGATGATGTGCAGCACTCCCAGTAGCT
AthiA1_08219/FN	ACCGCTAGCTAAGGAGGGTGAGTCATGACAGCACAATCAC
AthiA1_08219/RH	ACCAAGCTTCAATGATGATGATGATGATGATGATGAGGCAGCAGGCGC
AthiA1_08224/FN	ACCGCTAGCTAAGGAGGCATAAAAATGGAACAACTTCTCCAAA
AthiA1_08224/RH	ACCAAGCTTCAATGATGATGATGATGATGATGATGAGTGGATGAGTTTGCCTGA
AthiA1_10484/FN	ACCGCTAGCTAAGGAGGTGCAGCGATGAATAGCTCCCTT

Table S2. (Continued)

AthiA1_10484/RSa	ACCGTCGACCAATGATGATGATGATGATGATGATGATGCTGAGTACCATGGCTACAACT
37-53 F/NheI	ACCGCTAGCAGGAGGAATTCACCATGTACAATAAAGATGAT
37-53 R/SphI	ACCGCATGCTCAATGATGATGATGATGATGAGAAATCAATTGAATTTG
AthiA1_15675/FN	ACCGCTAGCTAAGGAGGGTCAACTATGCGACAGATGGCA
AthiA1_15675/RH	ACCAAGCTTCAATGATGATGATGATGATGATGATGATCTTTTTTTTCAGCCGCCAG
135-10 F/NheI	ACCGCTAGCAGGAGGAATTCACCATGTCCCGTGATGCTCCTG
135-10 R/SphI	ACCGCATGCTCAATGATGATGATGATGATGACTTTCATCTGCATCGCTC
40-113 F/NheI	ACCGCTAGCTAAGGAGGTGAGTAGATGGATAATCAATCCCCC
40-113 R/KpnI	ACCGGTACCTCAATGATGATGATGATGATGATGATGTGATATCTGACGCTTTTTGCTTTT
AthiA1_00152 F	CGTCCATTTATGACCCCAAC
AthiA1_00152 R	CGATCTTCTGCAGTCAACCA
AthiA1_00157 F	GCTTATCATCGCCAGGAATG
AthiA1_00157 R	CGAGCTAGATGACTTTGTCG
AthiA1_01972 F	GAAAGGGTACATCCTTGGAC
AthiA1_01972 R	GGTAGGATTATCGAGGATCGGC
AthiA1_01982 F	GAAAGGACAGCTCCATTACC
AthiA1_01982 R	GTAGATACATCGTCCGGTTCTG
AthiA1_03313 F	CAGCTGAAGCGAAAACATGA
AthiA1_03313 R	GAGGCATTCTTCGGCACTAC
AthiA1_04113 F	GAAAGACCTGCCTATTGACG
AthiA1_04113 R	CTTCAGTCTCTACACCCTCTAC
AthiA1_05539 F	CATTTGCGTAGACAGCCAGA
AthiA1_05539 R	ATGATCGCGAATTCATCTCC
AthiA1_06856 F	GTGACGGTTTCGGCAACTAT
AthiA1_06856 R	ACCAGCGGTTGATACCAAAG
AthiA1_07018 F	CCCGTCAATTACAGGGTATG
AthiA1_07018 R	GACCCGGAATAGACATCAAC
AthiA1_07707 F	ACCCTCAGGATCCCTGCTAT
AthiA1_07707 R	CATATAGGCAAAGGCGGAAA
AthiA1_08179 F	TTCGGCACGCAAAATGTTGGAC
AthiA1_08179 R	CTTCCAGCATATCGGCTTTGGGGTT
AthiA1_08184 F	CGATTACAGTCAGGGCTTCTAC
AthiA1_08184 R	CTTCTTCTATGTCACTGACGGG
AthiA1_08189 F	CGTAGTATCACTGAATGGGC
AthiA1_08189 R	CACTCAGCGAGTATATGGTG
AthiA1_08219 F	TTAATCGTCGCCGTATGGAG
AthiA1_08219 R	TAGTAGGGCGCAGCTTT
AthiA1_08224 F	ACGTCTCAATCAGCCAC
AthiA1_08224 R	GCCGGTCTTGAGCTTGAAATAA
AthiA1_09870 F	ACGCAGCAATCTCTTCTGGT
AthiA1_09870 R	AAGCAAGCTTTCACGCAAAT
AthiA1_09875 F	ACATCCTGCCAGCTTATTGG

Table S2. (Continued)

AthiA1_09875 R	ATGGGATGATCCTCTGTTGC
AthiA1_10484 F	CGAGTGGATTCATGTGAGTG
AthiA1_10484 R	GCCTCATCCAGAGTCAGACTATAC
AthiA1_12969 F	GGGTGTCGTGCTGTATCCTT
AthiA1_12969 R	ACGCTGATCTGCGAAGTTTT
AthiA1_12974 F	GACCGGGAGCTGATTATTGA
AthiA1_12974 R	ACATTCGTCGGGTACCTCTG
AthiA1_13196 F	ACCGAAACCGGATGTCATGT
AthiA1_13196 R	GCCAGAATGTCCTGACCAAT
AthiA1_13201 F	CTGGTCAAAATCGAGGTCGT
AthiA1_13201 R	TTCTCTTCCATCACGCACAG
AthiA1_14219 F	TGGCTGAGTTCATTGCTGAC
AthiA1_14219 R	TCGTCCAAATCCAGCATACA
AthiA1_14626 F	ATGCAGGATTTCCAGATTGC
AthiA1_14626 R	TGAGTATGTCCCGCACAAAA
AthiA1_15675 F	CTGACTCGTCCTACTGAGATT
AthiA1_15675 R	CAACATCTCACCACCAGAAC
AthiA1(13-20)F	TGGCCCGCTTGTATGATTT
AthiA1(13-20)R	TTTCGTAGCCTCCACAGTTATC
AthiA1(24-9)F	TACGATTTCAGTGCGAGTGG
AthiA1(24-9)R	GGTACCGCAGTTGGGTATTT
AthiA1(46-78b)F	TGCAGACGGGTGAAGTAATC
AthiA1(46-78b)R	AGCCTCTGGGAAACACTAAAT
AthiA1(83-30)F	TCCCGATGAGGTGGAAATTTAT
AthiA1(83-30)R	TTGGGACAGCGACTCATAATC
AthiA1(84-35)F	TATCCAGCGACCCGAAATG
AthiA1(84-35)R	TACAGCCCTTGCTCCATAAC
AthiA1(86-8)F	CTATCAAGGGTGGCGGATTAC
AthiA1(86-8)R	CCCAGATAACCTTACCCATCAC
AthiA1(115-12)F	TGGCTCGGGAAGACTATAAGA
AthiA1(115-12)R	ATCATACGCGGCACATCAA
Athia1(141-36)F	GAGATGCTGTTGGTGGAATTTG
Athia1(141-36)R	TAAGCATGGCTGGAATGGAG
AthiA1(FleQ1)F	ATGCGTCCTATGGATGGTTATAC
AthiA1(FleQ1)R	AGGGCTTCACCACATAATCTAC
AthiA1(FleQ2)F	CCGAAATGAGTCCGCAAATG
AthiA1(FleQ2)R	GAATCCGCTCCTCCAGATTAC
At_MshEN-F	TGCCCAGGAAGAGTTTGATTTA
At_MshEN-R	CACCCGGAAATCCTTCTCATAG
YajQ_F	AAGTAGATAACGCCCTGCATAC
YajQ_R	CTTTACCAGTCGGGCTGAAA

[1] Restriction sites are underlined.

Table S3. Type IV pilin-like protein and pili apparatus subunits encoding genes in *At. thioooxidans*^T^. Putative c-di-GMP effectors with PilZ domain are noted with grey background.

**Locus**[1]	**BLAST hit**
ATHIO_RS16400	type IV pilus assembly PilZ protein [*At. ferrooxidans* ATCC 53993]
ATHIO_RS0105675	type IV pilus assembly PilZ protein [*At. caldus* ATCC 51756]
ATHIO_RS0109125	type IV pilus assembly PilZ [*At. ferrivorans* SS3]
ATHIO_RS0110790	type IV pilus assembly PilZ [*At. caldus* ATCC 51756]
ATHIO_RS0114620	type IV pilus assembly PilZ protein [*At. ferrooxidans* ATCC 53993]
ATHIO_RS17100	prepilin IVa-type cleavage/methylation domain-containing protein [*At. thiooxidans* ATCC 19377]
ATHIO_RS0106075	prepilin-type cleavage/methylation domain-containing protein PilW [*At. thiooxidans* ATCC 19377]
ATHIO_RS0106080	prepilin-type cleavage/methylation domain-containing protein PilV [*At. thiooxidans* ATCC 19377]
ATHIO_RS0106085	prepilin-type cleavage/methylation domain-containing protein FimT [*At. thiooxidans* ATCC 19377]
ATHIO_RS0113585	prepilin IVa-type cleavage/methylation domain-containing protein [*At. thiooxidans* ATCC 19377]
ATHIO_RS0113590	prepilin IVa-type cleavage/methylation domain-containing protein [*At. thiooxidans* ATCC 19377]

[1] Gene code from NCBI**.**

Table S4. Putative molecular players for biofilm architecture identified in two iron/sulfur- (orange) and two sufur- (yellow) oxidizing species of *Acidithiobacillus* [a].

	Flagella	*Cellulose synthase*	*Pel exopolysaccharide*
At. ferrooxidans	-	-	-
At. ferrivorans	+	+	-
At. caldus	+	+	+
At. thiooxidans	+	+	+

[a], Based on this work and references [17, 25; 45]

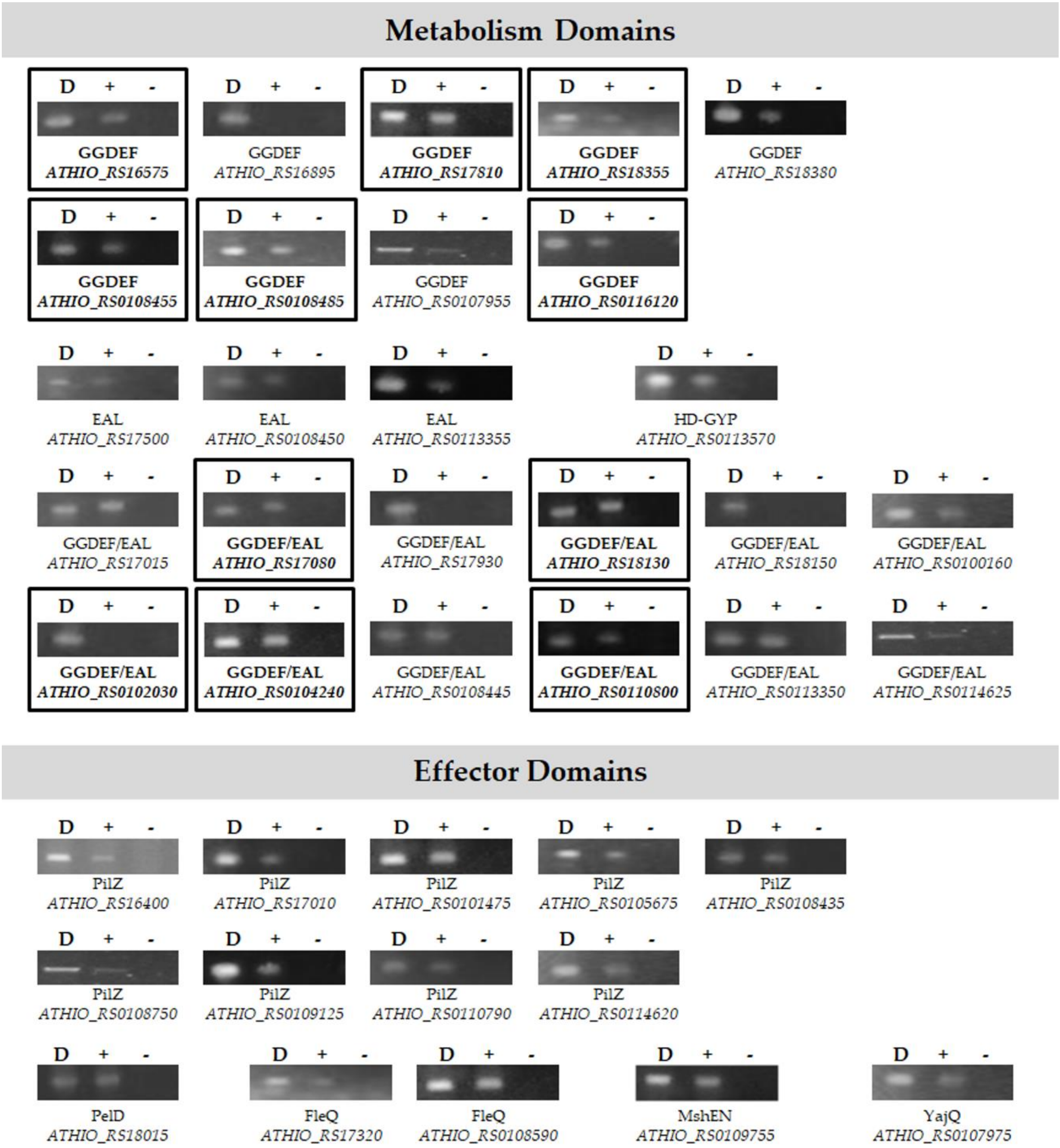

Figure S1. RT-PCR analysis of c-di-GMP metabolism and effectors encoding genes identified in *At. thiooxidans*^{T}. PCR assays were done with gDNA (D) and cDNA synthesized from total RNA in presence (+) or abscence of Reverse Transcriptase (-). Gene code was obtained from NCBI. Black boxes indicate ORFs tested for DGC activity.

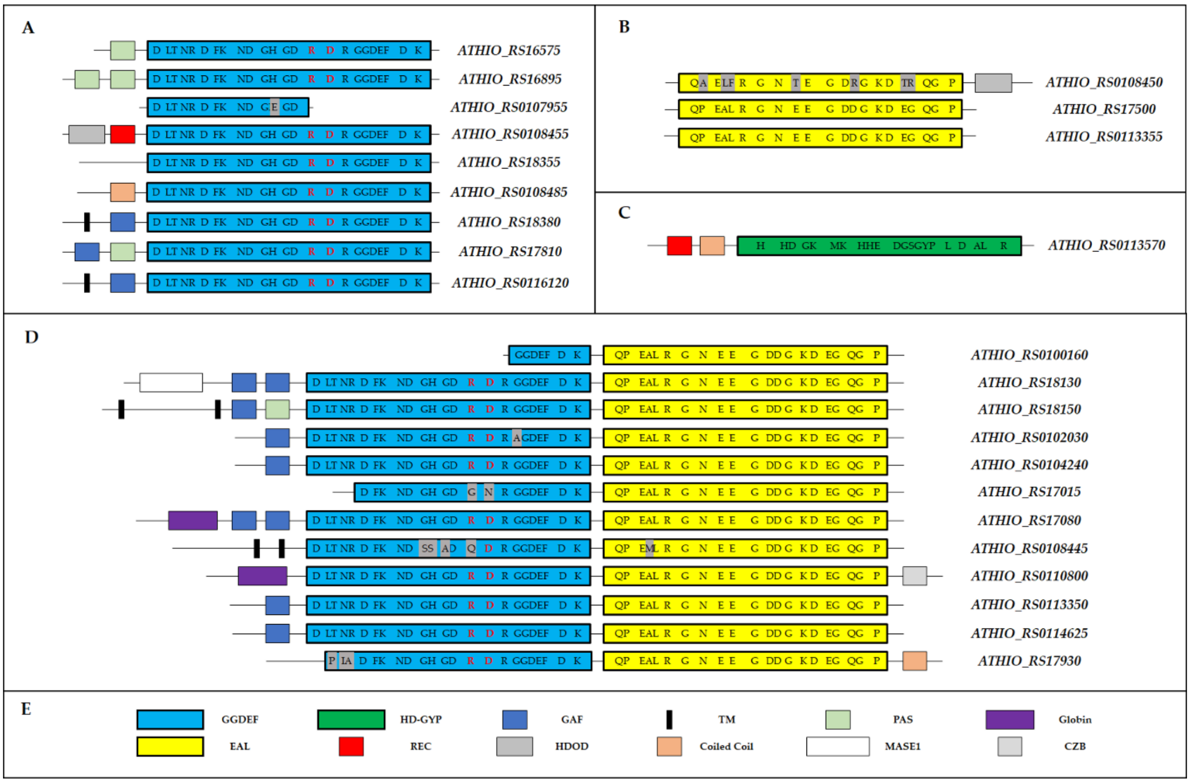

Figure S2. Domain organization of *At. thiooxidans*^{T} ATCC 19377 proteins involved in c-di-GMP metabolism. Schematic representations of single GGDEF- (**A**), single EAL- (**B**), single HD-GYP (**C**) and GGDEF/EAL- (**D**) domain containing proteins from *At. thiooxidans*^{T}. Color codes for the different domains are noted (**E**). Key aminoacids for catalytic activity are noted and non-conserved aminoacids are marked in grey. Domains are not drawn to scale. TM: transmembrane segment; CZB: chemoreceptor zinc-binding.

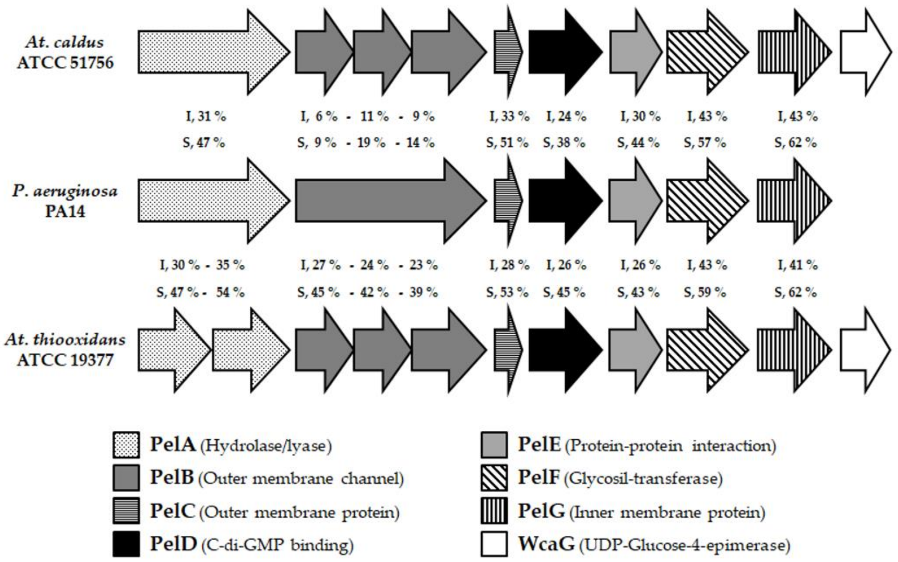

Figure S3. Comparative analysis of *pel* operon structures. Identity and similarity at protein level are noted. *Acidithiobacillus* sequences were obtained from NCBI genome database. Sequencing and/or annotation errors cannot be excluded for PelA and PelB proteins.

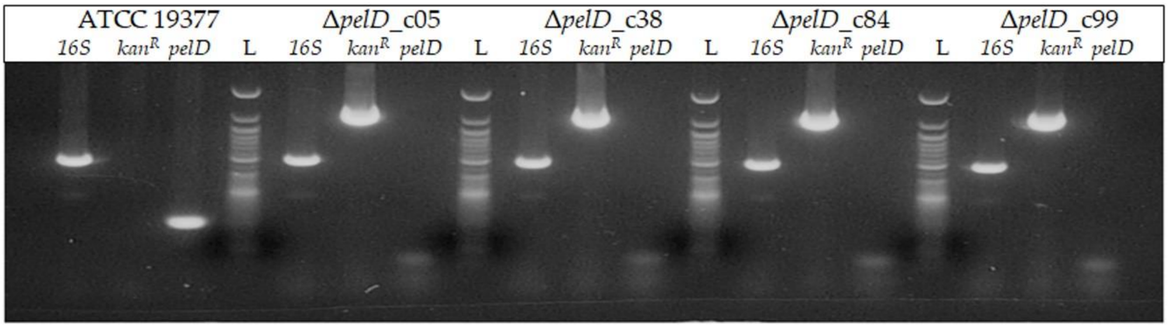

Figure S4. PCR analysis of *At. thiooxidans*^{T} wild type and the four double recombinant Δ*pelD* mutant strains to check double-recombination. DNAs genomic were analyzed with primers for *DNA 16S*, *kan*^{R} and *pelD* genes for 30 cycles. PCR products were run in 1 % agarose gel in TAE buffer 1X. L: DNA ladder. As expected wild type strain is positive for *DNA 16S* and *pelD* genes while double recombinant are negative for *pelD* gene and positive for *DNA 16S* and *kanR gene.*

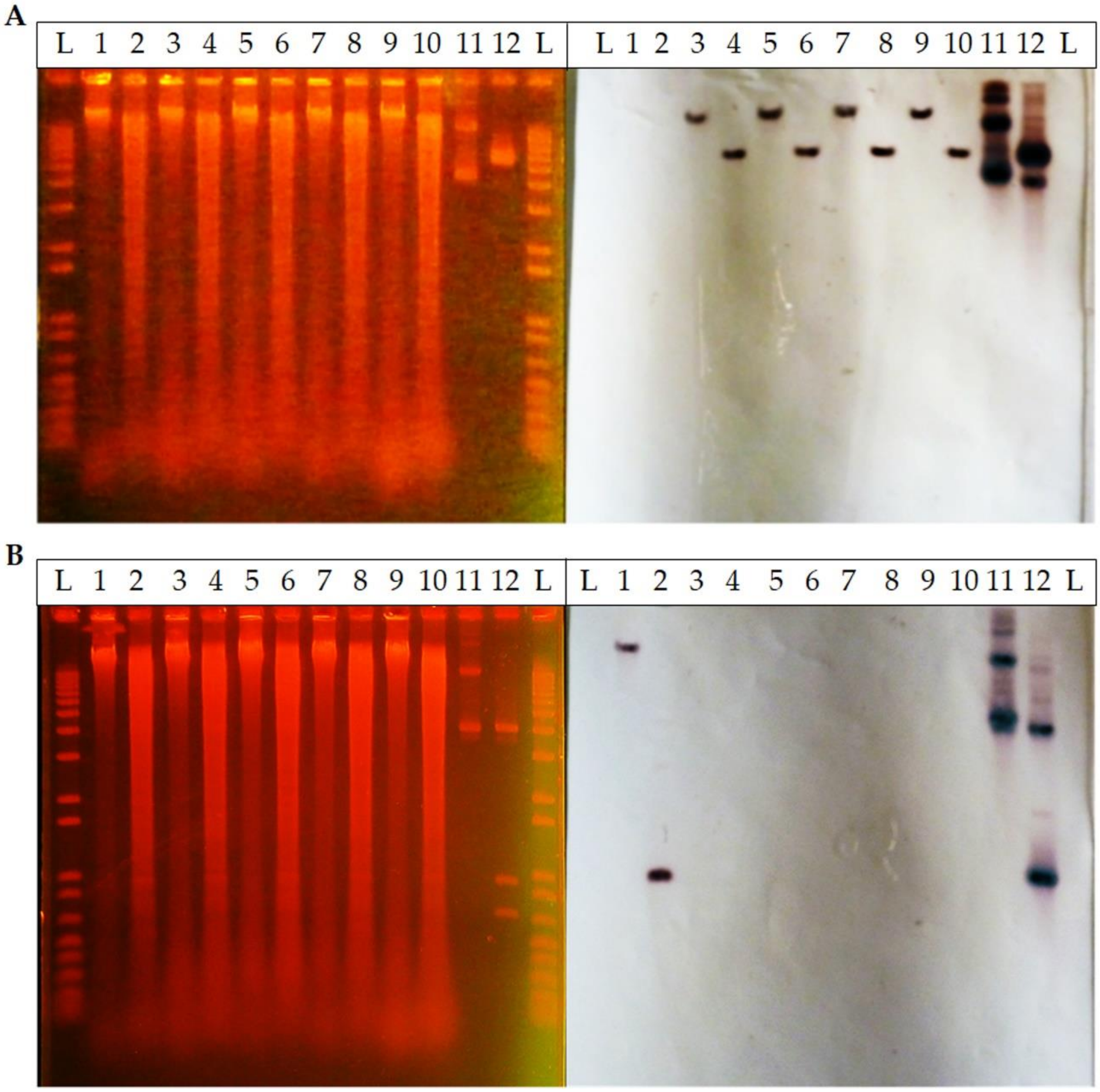

Figure S5. Southern Blot analysis of *At. thiooxidans*^{T} wild type and the four double recombinant Δ*pelD* mutant strains. 10 ◎g of genomic DNA no digested (odd numbers) or digested by *BamHI* (A) or *SphI* (B) (even numbers) were separated by running a DNA electrophoresis in a 1.5 % agarose gel and stained with GelRed® (left panels). Then DNA fragments were transferred onto nitrocellulose membranes for hybridization experiments with two specific digoxigenin-labelled DNA probes *kan*^{R} (A) and *pelD* (B). Positive results for *kan*^{R} and *pelD* probes correspond to 6-kb and 900-bp restriction fragments, respectively (red circles). L: DNA ladder; 1-2: ATCC 19377 DNA; 3-4: Δ*pelD_c05* 5-6: Δ*pelD_c38*; 7-8: Δ*pelD_c84;* 9-10: Δ*pelD_c99;* 11-12: positive control, pOT plasmids harboring *kan*^{R} (A) or *pelD* (B) genes.
